# Function and Pathological Implications of Exon Junction Complex Factor Y14

**DOI:** 10.3390/biom5020343

**Published:** 2015-04-10

**Authors:** Tzu-Wei Chuang, Kou-Ming Lee, Woan-Yuh Tarn

**Affiliations:** Institute of Biomedical Sciences, Academia Sinica, Taipei 115, Taiwan; E-Mails: tzuwei@ibms.sinica.edu.tw (T.-W.C.); g39002007@ym.edu.tw (K.-M.L.)

**Keywords:** exon-junction complex, nonsense-mediated mRNA decay, translation, pre-mRNA splicing, centrosome

## Abstract

Eukaryotic mRNA biogenesis involves a series of interconnected steps, including nuclear pre-mRNA processing, mRNA export, and surveillance. The exon-junction complex (EJC) is deposited on newly spliced mRNAs and coordinates several downstream steps of mRNA biogenesis. The EJC core protein, Y14, functions with its partners in nonsense-mediated mRNA decay and translational enhancement. Y14 plays additional roles in mRNA metabolism, some of which are independent of the EJC, and it is also involved in other cellular processes. Genetic mutations or aberrant expression of Y14 results in physiological abnormality and may cause disease. Therefore, it is important to understand the various functions of Y14 and its physiological and pathological roles.

## 1. Introduction

RNA processing is a critical step of eukaryotic gene expression and is tightly regulated to control and orchestrate a variety of biological processes. During transcription, nascent transcripts of RNA polymerase II undergo 5' capping, 3' cleavage, and polyadenylation as well as splicing, by which introns are removed, to become mature mRNAs. Throughout the maturation process, precursor mRNAs (pre-mRNAs) are decorated with various proteins to form a high-order messenger ribonucleoprotein (mRNP) complex. Mature mRNPs are exported to the cytoplasm where translation and mRNA turnover take place [1,2].

During splicing, a specific set of proteins that constitute the exon-junction complex (EJC) is loaded onto spliced mRNA [1,3]. The EJC is involved in several mRNA maturation events, including splicing, mRNA export, nonsense-mediated mRNA decay (NMD), and translational control, and it serves as an important molecular determinant of the nuclear history of mRNA (*i.e.*, influence of nuclear RNA processing events on the cytoplasmic fates of mRNAs) [4]. The tetrameric core of the EJC is composed of the Y14-Magoh heterodimer, eukaryotic initiation factor 4AIII (eIF4AIII), and metastatic lymph node 51 (MLN51, also known as Barentsz or CASC3). Deposition of the EJC core upstream of the spliced junction is mediated by the specific interaction between eIF4AIII and CWC22, a component of the spliceosome-activating PRP19 complex, during splicing [5,6]. In addition, several peripheral proteins join to form an outer shell of the EJC complex and participate in different steps of mRNA metabolism [7,8,9]. Among them, NXF1 acts as the primary mRNA export receptor, and the surveillance factors Upf3 and Upf2 function in NMD. NMD is an mRNA surveillance pathway that degrades aberrant mRNAs that contain a premature termination codon [10,11]. Upf3 and Upf2 recruit Upf1 to the EJC, which interacts with the upstream and halted ribosome on a premature termination codon and triggers degradation of the NMD-susceptible mRNA [12,13]. Otherwise, the EJC is removed from normal transcripts by the scanning ribosome during the pioneer round of translation [14].

Recent genome-wide analyses indicate that EJCs not only occupy the majority of exon junctions but also bind non-canonical sites in the coding sequences and the 5' and 3' untranslated regions (UTRs) [15,16,17]. Interestingly, EJC-binding sites are often associated with the RNA motifs that resemble the binding sites for serine-arginine-rich splicing factors (SR proteins). Therefore, it is possible that SR proteins can influence EJC loading to the sites beyond exon junctions. EJCs and SR proteins multimerize to promote packaging and compaction of spliced mRNAs and subsequent mRNA biogenesis steps [17]. In addition, individual EJC components can also exert distinct and EJC-independent functions in various cellular processes [18,19,20,21]. Furthermore, some genetic diseases are associated with mutations in the genetic loci encoding the EJC components [22]. In this article, we focus on various functions of the EJC core protein Y14 and its potential role in disease pathogenesis.

## 2. Y14 as an EJC Core Protein

Y14 and its heterodimeric partner Magoh are conserved from *Schizosaccharomyces pombe* to human. Y14 contains an RNA-binding domain (RBD) in the central region. Magoh binds with high affinity to the RBD surface of Y14 and masks its RNA-binding surface [23,24]. Notably, the most *C*-terminal region of vertebrate and *S. pombe* Y14 proteins contains two consecutive arginine/serine (RS) dipeptides. RS dipeptide phosphorylation can modulate the association of Y14 with other mRNA biogenesis factors and may govern its cellular functions [25]. For example, phosphorylated Y14 does not interact with the NMD factors, suggesting that phosphorylation of Y14 remodels the NMD complex during initial rounds of translation or prevents nonspecific NMD complex formation in the nucleus [25,26]. Moreover, the arginine residues adjacent to the RS dipeptides can be methylated, which is antagonized by RS dipeptide phosphorylation. Thus, the interplay between phosphorylation and methylation may modulate the various functions of Y14 [25].

In the EJC core, Y14 does not directly bind mRNA; instead eIF4AIII confers the activity for the sequence-independent interaction with exon junctions. In the presence of ATP, the two RecA domains of eIF4AIII form a large RNA clamp that interacts with the phosphate-ribose backbone of six nucleotides [27,28]. Y14-Magoh directly interacts with eIF4AIII and inhibits its ATPase activity to ensure the stable association of the EJC core with RNA [29]. The EJC core remains associated with the mRNA after its export to the cytoplasm [8]. Y14-Magoh is likely removed upon translation and subsequently recycled into the nucleus [14].

## 3. Subcellular Localization of Y14

Y14 localizes mainly in the nucleoplasm and continuously shuttles between the nucleus and the cytoplasm. Y14 may be imported into the nucleus alone or as a heterodimer with Magoh [30,31]. In the nucleus, Y14 as well as other EJC core factors, are concentrated in perispeckles, which surround splicing factor-enriched speckles [32]. The EJC core may assemble onto transcripts undergoing splicing in perispeckles [32]. In the cytoplasm, neither Y14-Magoh nor eIF4AIII is enriched in particular RNA granules, stress granules, or processing bodies (P-bodies) [21,33]. P-bodies are cytoplasmic foci containing translationally silenced mRNAs that are subject to degradation or reenter the translating pool [34]. Evidence indicates that some of the NMD factors cycle through P-bodies and accumulate in P-bodies when mRNA degradation is disrupted [35,36]. Localization of Y14 in cytoplasmic P-bodies is minimal but can be marginally enhanced by blocking disassembly of the NMD complex [35]. However, depletion of Y14 impedes P-body formation or accumulation, suggesting that Y14 plays an essential role in P-body biogenesis [37]. More interestingly, overexpression of a phosphomimetic Y14 mutant induces P-body formation [37]. Perhaps blockade of Y14 dephosphorylation prevents recycling of mRNA-degradation factors and in turn causes their accumulation in P-bodies. Nevertheless, this observation reiterates the importance of the phosphorylation/dephosphorylation cycle of Y14 in remodeling of post-splicing mRNPs and perhaps in determining mRNA fate.

## 4. Canonical Functions of Y14 in mRNA Metabolism

### 4.1. Y14 Plays an Important Role in NMD

Tethering of Y14 to the 3' UTR of NMD reporter mRNAs elicits their degradation [10,38,39,40]. Accordingly, depletion of Y14 impairs the degradation of premature termination codon-containing transcripts, suggesting a critical role of Y14 in NMD [38,39]. Y14-Magoh directly interacts with Upf3 to form an NMD-activating complex [11,41]. An early study indicated that Upf2 is required for tethered Y14-mediated NMD [38]. However, distinct branches of the NMD pathway have been proposed based on their different cofactor requirements and substrate features [39,41,42,43]. As compared with RNPS1-induced NMD, the Y14-Magoh-mediated pathway appears to depend to a lesser extent on Upf2, suggesting that Y14 has differential contributions across different NMD pathways [39,43]. Moreover, Y14 is also reported to be involved in fail-safe NMD, which requires the long 3' UTR and is generally EJC-independent [44]; whether the binding of Y14 to the 3' UTR and/or translational enhancement by Y14 (see below) contribute to the fail-safe NMD remains to be determined. Therefore, individual EJC factors may function via interactions with somewhat different sets of NMD factors to differentially determine the fate of individual mRNAs or act in response to particular cellular signaling pathways or environmental cues.

### 4.2. Y14 Enhances Translation

It has been long believed that splicing enhances mRNA expression, which occurs in part through the action of the EJC [45]. The EJC has a particular function in the cap-binding complex (CBC)-mediated pioneer round of translation, by which it enhances NMD [14,46]. eIF4AIII directly interacts with the CBC-dependent translation initiation factor (CTIF), which recruits the translation initiation factor eIF3 complex to facilitate the loading of the 40S ribosomal subunit onto the 5' end of an mRNA [20,47]. Y14-Magoh interacts with the partner of Y14-Magoh (PYM) protein, a factor that stimulates translation by bridging the EJC and 48S pre-initiation complex [48]. Tethering of either Y14 or Magoh to an intron-containing luciferase transcript results in higher translational yield [49]. Depletion of Y14 inhibits splicing-dependent translational activation, reinforcing its essential role in translation enhancement [50]. Similar effects have been detected for eIF4AIII. Moreover, overexpression of MLN51 can enhance productive translation [51]. However, an analysis using luciferase reporters containing different internal ribosome entry sites indicates that Y14 may function in an earlier step of translation, whereas eIF4AIII activates translation after 80S ribosome complex formation [50]. Perhaps EJC factors act in concert to promote the pioneer round of translation but modulate productive translation via different mechanisms and variably regulate specific genes. The finding that Y14 binds the mRNA 5' cap suggests an additional possibility that it might modulate translation by interacting with the cap [37].

## 5. Additional Functions of Y14 in Various Biological Processes

### 5.1. Y14 Promotes PRMT5 Activity

We have reported an unprecedented role for Y14 in promoting the activity of the methylosome [52]. The Y14-Magoh heterodimer specifically interacts with the cytoplasmic methylosome, which is composed of the arginine methyltransferase PRMT5, chaperon protein pICln, and WD repeat-containing protein MEP50/WD45 [53,54,55]. PRMT5 can symmetrically dimethylate arginine residues and target a wide range of substrates, including the Sm proteins of the spliceosomal small nuclear ribonucleoproteins (snRNPs) [56,57]. During snRNP biogenesis, the cytoplasmic survival of motor neuron (SMN) complex facilitates the assembly of methylated Sm proteins onto the snRNA. Y14 enhances PRMT5-mediated methylation of Sm proteins *in vitro* [52]. Overexpression of Y14 enhances Sm protein methylation and assembly by inducing the formation of a larger methylosome complex containing multimerized PRMT5, which confers higher methylation activity, and the SMN complex [52]. The finding that Y14 also participates in snRNP biogenesis may indicate that Y14 coordinates snRNP production with pre-mRNA splicing and maturation, and perhaps it may fine-tune mRNA expression.

### 5.2. Y14 Inhibits mRNA Decapping

Y14-Magoh, but not eIF4AIII or MLN51, specifically associates with the mRNA decapping complex and mRNA degradation factors, suggesting a specific and EJC-independent role for Y14 in modulating mRNA degradation [37]. Indeed, Y14 directly interacts with Dcp2 and inhibits the decapping activity of Dcp2 *in* *vitro*. Overexpression of Y14 prevents degradation of reporter mRNAs bearing an AU-rich destabilizing element [37]. It remains unclear, however, whether the binding of Y14 to the 5' cap is a prerequisite for its role in preventing mRNA decay. Notably, in regard to cap binding and mRNA stabilization, Y14 acts analogously to the testis-specific decapping regulator VCX-A (variably charged, X chromosome mRNA on CRI-S232A). VCX-A may function in brain development, and its deletion is associated with mental retardation [58,59]. Interestingly, Y14 has been detected in both the axons and dendrites of the primary cortical neurons [60]. Gain-of-function or overexpression of Y14 in the brain results in abnormal behavior [60,61]. Thus, it is of great interest to elucidate how Y14 may regulate mRNA surveillance or decay in EJC-dependent or -independent pathways in neurons.

### 5.3. Y14 Regulates Alternative Splicing

Y14 as well as several other EJC factors can directly regulate alternative splicing. This is not completely unexpected because the EJC core components are likely loaded onto pre-mRNA prior to completion of splicing and are indeed detected in late spliceosomal complexes [62,63]. Evidence indicates that EJC factors particularly regulate alternative splicing of apoptotic factors, which generate isoforms with opposing roles in apoptosis [9]. Depletion of Y14 enhances the expression of the proapoptotic isoforms of Bcl-x, Bim, and Mcl1, and accordingly promotes apoptosis. Y14 was also identified through a loss-of-function screen of genes involved in cell proliferation and apoptosis in a human mesothelioma cell line [64]. Therefore, Y14 may act as an anti-apoptotic factor and regulate cell viability. Notably, Magoh and certain other EJC factors specifically regulate alternative splicing of RAS/MAPK signaling factors in *Drosophila* [65,66]. However, whether and how EJC-mediated splicing regulation is functionally linked to NMD still warrants further investigation.

### 5.4. The Role of Y14 in Cell Cycle Control

Depletion of NMD factors impedes cell proliferation and causes cell cycle arrest [67,68,69]. Indeed, some of the NMD factors regulate the expression of gene sets associated with particular biological processes, including cell cycle progression [69]. Knockdown of individual EJC core factors results in mitotic spindle defects and an increase in DNA damage foci [18]. Notably, a mutagenesis screen determined the specific role of Magoh in mouse neural stem cell division as well as its contribution to efficient centrosome maturation in neural progenitor cells [18]. Depletion of Y14 results in G2/M arrest followed by apoptosis, but its overexpression also causes cell death [67,70]. It is possible that Y14-Magoh, in conjunction with other mRNA surveillance factors, particularly modulates the expression of cell cycle-related transcripts via splicing-coupled NMD, and thereby regulates cell viability. Coincidently, most recent reports have indicated that since a set of NMD targets encodes cell cycle inhibitory and pro-apoptotic factors, attenuation of NMD sensitizes cancer cells to chemotherapeutic agent-induced apoptosis [71,72]. Nevertheless, the observation that both Y14 and Magoh physically localize to the centrosome suggests their potential role in centrosome regulation [67]. The centrosome is essential not only for mitotic spindle formation but also for G2/M checkpoint control and DNA damage signaling [73]. Therefore, Y14 may be essential for mitotic progression or act as a regulator of this process.

## 6. The Physiological and Pathological Roles of Y14

### 6.1. Y14 in the Thrombocytopenia-Absent Radius Syndrome

Y14 has been implicated in the thrombocytopenia-absent radius (TAR) Syndrome [74,75]. TAR results from deletion of one *RBM8A* allele that encodes Y14 on chromosome 1q21.1 in conjunction with deleterious regulatory single-nucleotide polymorphisms in the other allele, and it is characterized by both thrombocytopenia and absent radii with the presence of thumbs [74,75]. In particular, TAR patients have low numbers of platelet precursors in the bone marrow. This is reminiscent of a previous study showing that hematopoietic-specific depletion of Upf2 resulted in depletion of hematopoietic stem cells [76]. Thus, Y14 as well as some of the NMD factors may particularly regulate the expression of genes involved in the proliferation of hematopoietic cells. Moreover, deletions of the 1q21.1 proximal region are associated with a spectrum of developmental delays, particularly in the brain [77], suggesting that *RBM8A* and/or adjacent genes may play roles in brain development.

### 6.2. Y14 in Neuronal Development and Function

The finding that Y14-deficient TAR patients exhibit mental retardation and Y14 is expressed in neurons suggests a role for Y14 in neuronal development and function [18,77,78]. Additionally, copy number variations of several EJC/NMD factors including Y14 also lead to neurological diseases [61]. Overexpression of Y14 in the dentate gyrus of mice results in abnormal emotional behavior [60]. Accordingly, Y14 binds the mRNA transcripts that impact synaptic plasticity and behavior, and its overexpression affects miniature excitatory postsynaptic currents in cultured neurons, suggesting increased synaptic contacts [60]. A mutagenesis screen revealed that Magoh haploinsufficiency causes microcephaly owing to depletion of intermediate neural progenitors and neuronal apoptosis [18]. Magoh is involved in neural stem cell division and controls the expression level of the microcephaly-associated protein Lis1 during neurogenesis [18]. It is noteworthy that MagohB, the Magoh analog, may function redundantly with Magoh, but their expression may be differentially regulated [79]. Moreover, microdeletions of chromosome regions encompassing *RBM8A* are associated with mental retardation and microcephaly [77,80], emphasizing the role of Y14-Magoh in neuronal development. The observation that Y14 or Magoh depletion disrupts mitotic spindle orientation and integrity, chromosome number, and genomic stability may explain their possible role in neurogenesis during brain development [18]. Perhaps the potential roles of Y14 and Magoh in centrosomal function also contribute to proper neuronal cell division during development.

### 6.3. Additional Pathological Potential of Y14

Autoantibodies against Sm proteins are prevalent in patients with systemic lupus erythematosus. The anti-Sm autoimmune response involves multiple epitopes of Sm proteins, including symmetrically dimethylated arginines in the arginine-glycine repeats of Sm D1 and D3 [81,82]. Differential methylation of Sm proteins may account for the observed variations in autoantibody expression and affinity [82,83]. Therefore, we speculate that Y14 may modify immune function in TAR patients via PRMT5-mediated protein methylation. Furthermore, Y14 inhibits mRNA decapping and modulates alternative splicing, which conceivably would impact the transcriptome profile and have physiological consequences.

## 7. Conclusions and Prospective

Y14 is engaged in several steps of mRNA biogenesis and translation and may also have specialized roles in other cellular processes such as signaling and centrosome control [19,21,35]. Questions listed below remain to be answered by future investigation.

Accumulating evidence indicates that various NMD targeting features exist, such as an atypically long 3' UTR and upstream open reading frame [22]. Notably, EJCs also bind to UTRs, *i.e.*, non-canonical binding sites for the EJC [15,16,20]. Therefore, it will be interesting to know whether those non-canonical NMD events exhibit different dependency on the EJC complex or involve variable EJC components. Moreover, a recent report showed that eIF4AIII binds to the 5' UTR of transcripts and facilitates the translation of those transcripts containing structured elements [20]. Perhaps Y14 also has preferential targets and modulates their expression via diverse mechanisms.

Recent studies have begun to reveal the effect of cellular signaling pathways on NMD. Under nutrient-sufficient conditions, the mTOR-activated kinase S6K1 is recruited to the EJC bound to newly synthesized mRNAs to promote their pioneer round of translation [84]. In stressed cells, NMD is generally inhibited, but selective translation may be maintained for cellular adaptation and to ensure cell survival [85,86]. Questions remain as to whether any of the EJC components are modified and how the activity of the EJC is modulated by various cellular stimuli. Our previous report showed that phosphorylation/dephosphorylation of Y14 modulates its interaction with the NMD and translation factors [25]. Thus, Y14 may be a target of cellular signaling kinases. Nevertheless, this hypothesis as well as the specific function of Y14 in response to cellular cues remains to be tested.

EJC and NMD factors are particularly engaged in the control of cell cycle progression and maintenance of genome stability [22,76]. As intriguing as the centrosomal localization of Y14-Magoh, several NMD effectors, namely suppressors with morphogenetic defects in genitalia (SMG) proteins, are enriched at telomeres [87]. Notably, centrosomal components also include RNA, which has been proposed to interact with spindles in a structural role or to determine asymmetric centrosome localization during stem cell division [88]. It would be of interest to know whether Y14 and Magoh function as mitotic regulators via association with centrosomal RNAs and/or participate in local mRNA translation at the centrosome.

Finally, the implication of EJC and NMD factors in neurodevelopmental disorders is also noteworthy [22,89,90]. NMD is coupled to alternative splicing and eliminates aberrant splicing products to ensure proper gene expression. The abundance of alternative splicing events that take place in neuronal cells may render them sensitive to NMD. Nevertheless, the specific role of Y14 in neuronal mRNA expression and perhaps in neuronal stem cell division warrants future investigation.
